# Supplementary material for: Altered microRNA Transcriptome in Cultured Human Airway Cells upon Infection with SARS-CoV-2
Source: Viruses. 2023 Feb 10;15(2):496. doi: 10.3390/v15020496 (PMC9962802; doi:10.3390/v15020496)

Supplementary file

Experiment Workflow

RNA Sample QC

The purity and concentration of total RNA samples were determined with NanoDrop ND-1000. Results were provided in Sample QC report.

Sequencing Library Preparation

Total RNA of each sample was sequentially ligated to 3' and 5' small RNA adapters. cDNA was then synthesized and amplified using Illumina's proprietary RT primers and amplification primers. Subsequently, ~130 -150 bp PCR amplified fragments were extracted and purified from the PAGE gel. And finally, the completed libraries were quantified by Agilent 2100 Bioanalyzer.

Cluster Generation

The samples were diluted to a final concentration of 8 pM and cluster generation was performed on the Illumina cBot using TruSeq Rapid SR cluster kit (#GD-402-4001, Illumina), following manufacturer's instructions.

Sequencing on Illumina NextSeq 500

Sequencing was performed on Illumina NextSeq 500 using TruSeq Rapid SBS Kits (#FC-402-4002, Illumina), according to the manufacturer's instructions

Data Collection and Analysis

Supplementary file

Flowchart of Data Analysis

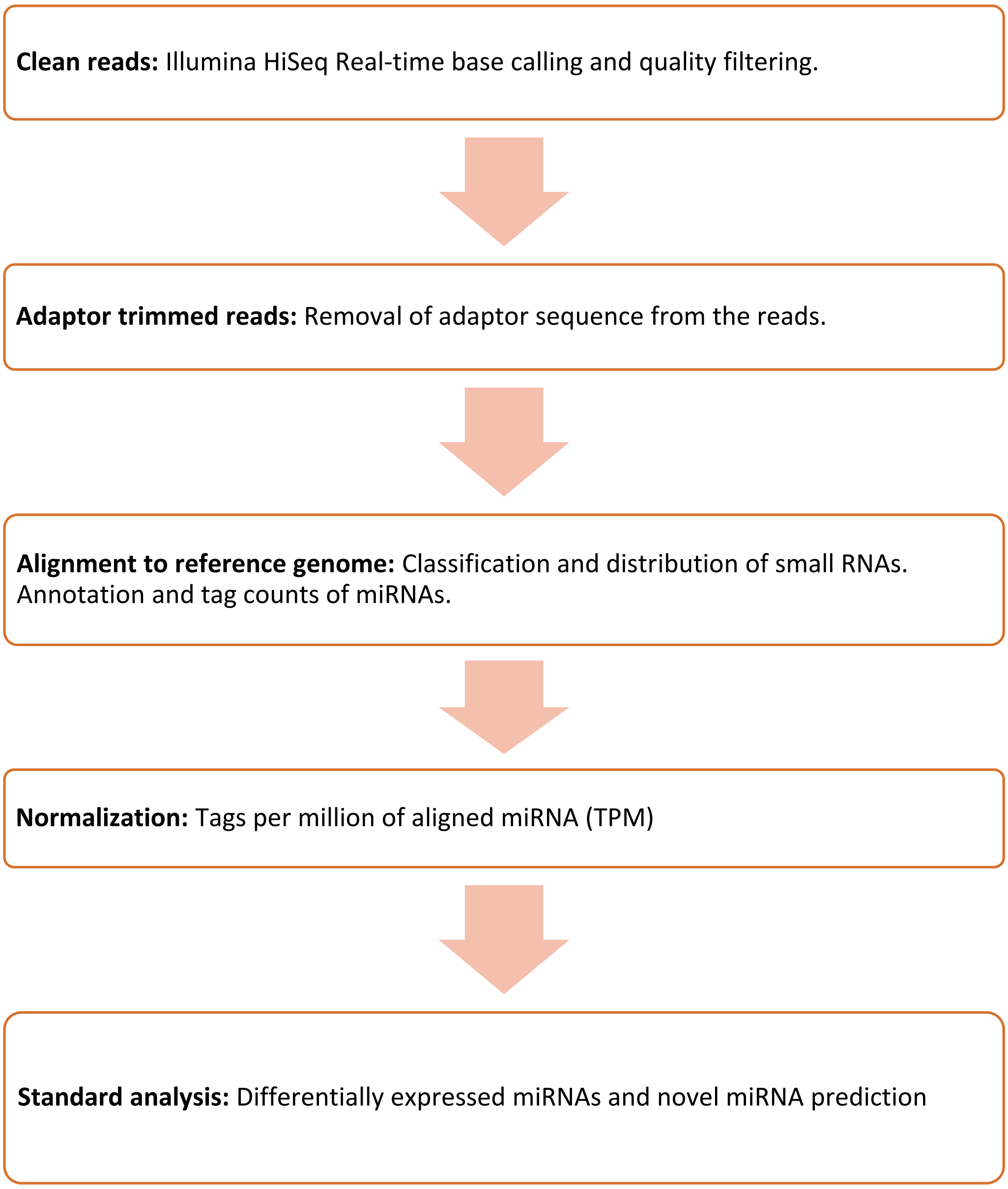

Supplement: Supplementary file 1 [file viruses-15-00496-s001.zip › viruses-2130726 Suppmentary File S1-S4/Supplementary File S1_ RNA-Seq_Workflow/Experiment_Workflow_and_Flowchart_of_Data_Analysis.pdf]
