# Supplementary material for: Altered microRNA Transcriptome in Cultured Human Airway Cells upon Infection with SARS-CoV-2
Source: Viruses. 2023 Feb 10;15(2):496. doi: 10.3390/v15020496 (PMC9962802; doi:10.3390/v15020496)
Supplement: Supplementary file 1 [file viruses-15-00496-s001.zip › viruses-2130726 Suppmentary File S1-S4/Supplementary FIle S4_ plasmid_construction/3'UTR_ACE2_WT_IDT.pdf]

INTEGRATED DNA TECHNOLOGIES

OLIGONUCLEOTIDE SPECIFICATION SHEET

01-Nov-2021

Sales Order Number **18069117**

Reference Number **316213144**

Customer Name - IDRISSA DIALLO

Name - 3-UTR-ACE2_WT

gBlocks® Gene Fragments 898 base pairs

```
5'- TAA GCA CTC GAG AAA AAT CTA TGT TTT TCC TCT TGA GGT GAT TTT GTT GTA TGT AAA TGT TAA TTT CAT
GGT ATA GAA AAT ATA AGA TGA TAA AGA TAT CAT TAA ATG TCA AAA CTA TGA CTC TGT TCA GAA AAA AAA TTG
TCC AAA GAC AAC ATG GCC AAG GAG AGA GCA TCT TCA TTG ACA TTG CTT TCA GTA TTT ATT TCT GTC TCT GGA
TTT GAC TTC TGT TCT GTT TCT TAA TAA GGA TTT TGT ATT AGA GTA TAT TAG GGA AAG TGT GTA TTT GGT CTC
ACA GGC TGT TCA GGG ATA ATC TAA ATG TAA ATG TCT GTT GAA TTT CTG AAG TTG AAA ACA AGG ATA TAT CAT
TGG AGC AAG TGT TGG ATC TTG TAT GGA ATA TGG ATG GAT CAC TTG TAA GGA CAG TGC CTG GGA ACT GGT GTA
GCT GCA AGG ATT GAG AAT GGC ATG CAT TAG CTC ACT TTC ATT TAA TCC ATT GTC AAG GAT GAC ATG CTT TCT
TCA CAG TAA CTC AGT TCA AGT ACT ATG GTG ATT TGC CTA CAG TGA TGT TTG GAA TCG ATC ATG CTT TCT TCA
AGG TGA CAG GTC TAA AGA GAG AAG AAT CCA GGG AAC AGG TAG AGG ACA TTG CTT TTT CAC TTC CAA GGT GCT
TGA TCA ACA TCT CCC TGA CAA CAC AAA ACT AGA GCC AGG GGC CTC CGT GAA CTC CCA GAG CAT GCC TGA TAG
AAA CTC ATT TCT ACT GTT CTC TAA CTG TGG AGT GAA TGG AAA TTC CAA CTG TAT GTT CAC CCT CTG AAG TGG
GTA CCC AGT CTC TTA AAT CTT TTG TAT TTG CTC ACA GTG TTT GAG CAG TGC TGA GCA CAA AGC AGA CAC TCA
ATA AAT GCT AGA TTT ACA CAC TCG CGG CCG CAT TCG T -3'
```

Note 1: The sequence information of the sense strand displayed above is intentionally truncated to the first 1,200 bases. The complete sequence can be verified in FASTA format in your order history.

Note 2: gBlocks® Gene Fragments are delivered as double-stranded DNA. Conformance to quality standards is established in multiple ways, including size verification by capillary electrophoresis and sequence identification by mass spectrometry.

Properties

Length:	898
Amount Delivered:	1000ng
GC Content:	38.86%
Molecular Weight:	554650.2
fmoles/ng:	1.80
µg/OD_{260}:	50
Normalization	1000ng = 1802fmol, dried

316213144 IDT
IDRISSA DIALLO
456197084 **01-Nov-2021**
gBlocks® Gene Fragments
3-UTR-ACE2_WT
1000ng = 1802fmol, dried

316213144 IDT
IDRISSA DIALLO
456197084 **01-Nov-2021**
gBlocks® Gene Fragments
3-UTR-ACE2_WT
1000ng = 1802fmol, dried

Instructions

Dried contents may appear as either a translucent film or a white powder. This variance does not affect the quality of the gBlocks® Gene Fragments.

Resuspending your gBlocks® Gene Fragments
1. Prior to opening, centrifuge the tube at a minimum of 3000 x g to ensure that the material is at the bottom of the tube.
2. Add TE to reach a final concentration of 10ng/µL.
3. Vortex briefly.
4. Incubate at 50°C for 20 minutes.
5. Briefly vortex and centrifuge.

Amplifying your gBlocks® Gene Fragments
•For gBlocks® Gene Fragments ≤1kb, amplification can be performed using a high fidelity polymerase. To avoid sequence mutations due to amplification errors limit cycles to 12 or fewer.
•For gBlocks® Gene Fragments >1kb, we do not recommend amplification.

For additional information please see www.idtdna.com/gblockssupport or contact genes@idtdna.com

Disclaimer

See page notes (I), (II), & (III) on reverse for usage, label license, and product warranties

I. Use Restrictions: Oligonucleotides and nucleic acid products are manufactured and sold by IDT for the customer's research purposes only. Resale of IDT products requires the express written consent of IDT. Unless pursuant to a separate signed agreement by authorized IDT officials, IDT products are not sold for (and have not been approved) for use in any clinical, diagnostic or therapeutic applications. Obtaining license or approval to use IDT products in proprietary applications or in any non-research (clinical) applications is the customer's exclusive responsibility. *IDT will not be responsible or liable for any losses, costs, expenses, or other forms of liability arising out of the unauthorized or unlicensed use of IDT products. Purchasers of IDT products shall indemnify and hold IDT harmless for any and all damages and/or liability, however characterized, related to the unauthorized or unlicensed use of IDT products. Under no circumstances shall IDT be liable for any consequential damages, resulting from any use (approved or otherwise) of IDT products.* All orders received by IDT, and all sales of IDT products are made subject to the aforementioned use restrictions and customer indemnification of IDT.

II. General Warranty: IDT's products are guaranteed to meet or exceed our published specifications for identity, purity and yield as measured under normal laboratory conditions. If our product fails to meet such specifications, IDT will promptly replace the product. *All other warranties are hereby expressly disclaimed, including but not limited to, the implied warranties of merchantability and fitness for a particular purpose, and any warranty that the products, or the use of products, manufactured by IDT will not infringe the patents of one or more third-parties*All orders received by IDT, and all sales of IDT products are made subject to the aforementioned disclaimers of warranties.

III. See http://www.idtdna.com/Catalog/Usage/Page1.aspx for further details

a) Cy Dyes: The purchase of this Product includes a limited non-exclusive sublicense under U.S Patent Nos. 5 556 959 and 5 808 044 and foreign equivalent patents and other foreign and U.S counterpart applications to use the amidites in the Product to perform research. NO OTHER LICENSE IS GRANTED EXPRESSLY, IMPLIEDLY OR BY ESTOPPEL. *Use of the Product for commercial purposes is strictly prohibited without written permission from Amersham Biosciences Corp.* For information concerning availability of additional licenses to practice the patented methodologies, please contact Amersham Biosciences Corp, Business Licensing Manager, Amersham Place, Little Chalfont, Bucks, HP79NA.

b) BHQ: Black Hole Quencher, BHQ-0, BHQ-1, BHQ-2 and BHQ-3 are registered trademarks of Biosearch Technologies, Inc., Novato, California, U.S.A Patents are currently pending for the BHQ technology and such BHQ technology is licensed by the manufacturer pursuant to an agreement with BTI and these products are sold exclusively for research and development use only. They may not be used for human veterinary in vitro or clinical diagnostic purposes and they may not be re-sold, distributed or re-packaged. For information on licensing programs to permit use for human or veterinary in vitro or clinical diagnostic purposes, please contact Biosearch at licensing@biosearchtech.com.

c) MPI dyes: MPI dyes. This product is provided under license from Molecular Probes, Inc., for research use only, and is covered by pending and issued patents.

d) Molecular Beacons: Molecular Beacons. This product is sold under license from the Public Health Research Institute only for use in the purchaser’s research and development activities.

e) ddRNAi: This product is sold solely for use for research purposes in fields other than plants. This product is not transferable. If the purchaser is not willing to accept the conditions of this label license, supplier is willing to accept the return of the unopened product and provide the purchaser with a full refund. However if the product is opened, then the purchaser agrees to be bound by the conditions of this limited use statement. This product is sold by supplier under license from Benitec Australia Ltd and CSIRO as co-owners of U.S Patent No. 6,573,099 and foreign counterparts. For information regarding licenses to these patents for use of ddRNAi as a therapeutic agent or as a method to treat/prevent human disease, please contact Benitec at licensing@benitec.com. For the use of ddRNAi in other fields, please contact CSIRO at www.pi.csiro.au/RNAi.

f) Dicer Substrate RNAi:

- These products are not for use in humans or non-human animals and may not be used for human or veterinary diagnostic, prophylactic or therapeutic purposes. Sold under license of patents pending jointly assigned to IDT and the City of Hope Medical Center.
- This product is licensed under European Patents 1144623, 121945 and foreign equivalents from Alnylam Pharmaceuticals, Inc., Cambridge, USA and is provided only for use in academic and commercial research whose purpose is to elucidate gene function, including research to validate potential gene products and pathways for drug discovery and development and to screen non-siRNA based compounds (but excluding the evaluation or characterization of this product as the potential basis for a siRNA based drug) and not for any other commercial purposes. Information about licenses for commercial use (including discovery and development of siRNA-based drugs) is available from Alnylam Pharmaceuticals, Inc., 300 Third Street, Cambridge MA 02142, USA
- License under U.S. Patent # 6506559; Domestic and Foreign Progeny; including European Patent Application # 98964202

g) LNAs: Protected by US. Pat No. 6,268,490 and foreign applications and patents owned or controlled by Exiqon A/S. For Research Use Only. Not for resale or for therapeutic use or use in humans

h) Other siRNA duplexes: This product is provided under license from Molecular Probes, Inc., for research use only, and is covered by pending and issued patents.

i) Acrydite: IDT is licensed under U.S Patent Number 6,180,770 and 5,932,711 to sell this product for use solely in the purchaser's own life sciences research and development activities. Resale, or use of this product in clinical or diagnostic applications, or other commercial applications, requires separate license from Mosaic, Inc.

j) Iso-Bases: Licensed under EraGen, Inc. United States Patents Number 5,432,272; 6,001,983; 6,037,120; and 6,140,496. For research use Only.

k) Dig: Licensed from Roche Diagnostics GmbH

l) 5' Nuclease Assay: The 5' Nuclease Assay and other homogenous amplification methods used in connection with the Polymerase Chain Reaction (PCR) process are covered by patents owned by Roche Molecular Systems, Inc. and F. Hoffman La-Roche Ltd (Roche). No license to use the 5' Nuclease Assay or any Roche patented homogenous amplification process is conveyed expressly or by implication to the purchaser by the purchase of the above listed products or any other IDT products.

m) Iowa Black® FQ and RQ: Iowa Black is a registered trademark of IDT, and Iowa Black-labeled oligos are covered by pending patents owned and controlled by IDT.

n) IRDye® 700 and IRDye® 800: IRDye® 700 and IRDye® 800 are products manufactured under license from LI-COR® Biosciences, which expressly excludes the right to use this product in qPCR or AFLP applications.
