# Supplementary material for: Altered microRNA Transcriptome in Cultured Human Airway Cells upon Infection with SARS-CoV-2
Source: Viruses. 2023 Feb 10;15(2):496. doi: 10.3390/v15020496 (PMC9962802; doi:10.3390/v15020496)
Supplement: Supplementary file 1 [file viruses-15-00496-s001.zip › viruses-2130726 Suppmentary File S1-S4/Supplementary FIle S4_ plasmid_construction/3_UTR_ACE2_Seed_miRNAs_seed.pdf]

>3’UTR-ACE2 (875nt) – Wild type

TAGAAAAATCTATGTTTTTCCTCTTGAGGTGATTTTGTTGTATGTAAATGTTAATTTCATGGTATAGAAAATATAAGATGATAAAGATA

TCATTAAATGTCAAAACTATGACTCTGTTCAGAAAAAAAAT**TGTCCAAAGAC**AACATGGCCAAGGAGAGAGCATCTTCATTGACATTGC

TTTCAGTATTTATTTCTGTCTCTGGATTTGACTTCTGTTCTGTTTCTTAATAAGGATTTTGTATTAGAGTATATTAGGGAAAGTGTGTA

TTTGGTCTCACAG**GCTGTTCAGGGATAATCTAA**ATGTAAATGTCTGTTGAATTTCTGAAGTTGAAAACAAGGATATATCATTGGAGCAA

GTGTTGGATCTTGTATGGAATATGGATGGATCACTTGTAAGGACAGTGCCTGGGAACTGGTGT**AGCTGCAAGGATTGAG**AATGGCATGC

ATTAGCTCACTTTCATTT**AATCCATT**GTCAAGGATGACATGCTTTCTTCACAGTAACTCAGTTCAAGTACTATGGTGATTTGCCTACAG

TGATGTTTGGAATCGATCATGCTTTCTTCAAGGTGAC**AGGTCTAAAGAG**A**GAAGAATCCAGG**GAACAGGTAGAGGACATTGCTTTTTCA

CTTCCAAGGTGCTTGATCAACATCTCCCTGACAACACAAAACTAGAGCCAGGGGCCTCCGTGAACTCCCAGAGCATGCCTGATAGAAAC

TCATTTCTACTGTTCTCTAACTGTGGAGTGAATGGAAATTCCAACTGTATGTTCACCCTCTGAAGTGGGTACCCAGTCTCTTAAATCTT

TTGTATTTGCTCACAGTGTTTGAGCAGTGCTGAGCACAAAGCAGACACTCAATAAATGCTAGATTTACACACTC

//

>3’UTR-ACE2 (875nt) – Mutant (MicroRNA Response éléments targeted by miR-1246)

TAGAAAAATCTATGTTTTTCCTCTTGAGGTGATTTTGTTGTATGTAAATGTTAATTTCATGGTATAGAAAATATAAGATGATAAAGATA

TCATTAAATGTCAAAACTATGACTCTGTTCAGAAAAAAAAT**TGTCCAAAGAC**AACATGGCCAAGGAGAGAGCATCTTCATTGACATTGC

TTTCAGTATTTATTTCTGTCTCTGGATTTGACTTCTGTTCTGTTTCTTAATAAGGATTTTGTATTAGAGTATATTAGGGAAAGTGTGTA

TTTGGTCTCACAG**GTTATCCGGAGGTGACCCAG**ATGTAAATGTCTGTTGAATTTCTGAAGTTGAAAACAAGGATATATCATTGGAGCAA

GTGTTGGATCTTGTATGGAATATGGATGGATCACTTGTAAGGACAGTGCCTGGGAACTGGTGT**GGTTACGAAGGTCGGG**AATGGCATGC

ATTAGCTCACTTTCATTT**TACCGACTGCC**GTCAAGGATGACATGCTTTCTTCACAGTAACTCAGTTCAAGTACTATGGTGATTTGCCTA

CAGTGATGTTTGGAATCGATCATGCTTTCTTCAAGGTGAC**AGGTCTAAAGAG**A**GAAGAATCCAGG**GAACAGGTAGAGGACATTGCTTTT

TCACTTCCAAGGTGCTTGATCAACATCTCCCTGACAACACAAAACTAGAGCCAGGGGCCTCCGTGAACTCCCAGAGCATGCCTGATAGA

AACTCATTTCTACTGTTCTCTAACTGTGGAGTGAATGGAAATTCCAACTGTATGTTCACCCTCTGAAGTGGGTACCCAGTCTCTTAAAT

CTTTTGTATTTGCTCACAGTGTTTGAGCAGTGCTGAGCACAAAGCAGACACTCAATAAATGCTAGATTTACACACTC

miR-1246:

- mfe: -14.8 kcal/mol - Position: 582
- mfe: -17.7 kcal/mol - Position: 417
- mfe: -18.0 kcal/mol- Position: 278
- Sequences in yellow: seed of miR-1246 reported in the literature (PMID: 28386354; PMID: 32432483) but also on TargetScan.

miR-1290 :

- mfe: -15.1 kcal/mol - Position: 569
- mfe: -14.5 kcal/mol - Position: 582
- mfe: -14.0 kcal/mol - Position: 128
