# Supplementary material for: Altered microRNA Transcriptome in Cultured Human Airway Cells upon Infection with SARS-CoV-2
Source: Viruses. 2023 Feb 10;15(2):496. doi: 10.3390/v15020496 (PMC9962802; doi:10.3390/v15020496)
Supplement: Supplementary file 1 [file viruses-15-00496-s001.zip › viruses-2130726-Supplementary_figures and tables.pdf]

Altered microRNA transcriptome in cultured human airway cells upon infection with SARS-CoV-2

Idrissa Diallo[1], Rajesh Abraham Jacob[2], Elodie Vion[1], Robert A. Kozak[3], Karen Mossman[2] and Patrick Provost[1,*]

[1] CHU de Québec-Université Laval Research Center/CHUL Pavilion, Department of Microbiology, Infectious Diseases and Immunology, Faculty of Medicine, Université Laval, Quebec City, QC, G1V 0A6, Canada.

[2] McMaster Immunology Research Centre, M.G. DeGroote Institute for Infectious Disease Research, Department of Medicine, McMaster University, Hamilton, ON L8S 4K1, Canada.

[3] Division of Microbiology, Department of Laboratory Medicine & Molecular Diagnostics, Sunnybrook Health Sciences Centre, Toronto, ON M4N 3M5, Canada.

The first two authors contributed equally to this work.

* Corresponding author: Dr. Patrick Provost

Phone: 1 418 525 4444 (ext. 48842)

E-mail: patrick.provost@crchudequebec.ulaval.ca

SUPPLEMENTARY FIGURES

In order of appearance in the manuscript

Table S1: The read counts at the data processing stages. The total number of reads (cleaned after 3′ adapter trimming and passed Solexa CHASTITY quality filter) at different sequencing data processing stages were averaged (n=3 biological replicates) and listed for each sample. Adapter-trimmed reads were expressed in counts and as percentage relative to all clean reads (8-30nt). Reads aligned to known human pre-miRNAs were expressed in counts and as percentage relative to the adapter-trimmed reads.

	Clean Reads	Adapter-trimmed Reads >= 16nt (% in clean reads)	Reads aligned to known pre-miRNA in miRBase21 (% in adapter-trimmed reads>16nt)
**Mock 24**	6469770	2632761 (41%)	721864 (27%)
**SARS-CoV-2 24h**	6026937	4701736 (78%)	1849304 (39%)
**Mock 72h**	6908146	4522348 (65%)	2434309 (54%)
**SARS-CoV-2 72h**	7004440	4842784 (69 %)	1924870 (40%)

Table S2. List of primers used in this study.

Genes	Sequences (5’-3’)	Accessions
**FW_ACE2**	GGGATCAGAGATCGGAAGAAGAAA	NM_001371415.1
**RV_ACE2**	AGGAGGTCTGAACATCATCAGTG	
**FW_TMPRSS2**	AGGTGAAAGCGGGTGTGAGG	NM_001135099.1
**RV_TMPRSS2**	ATAGCTGGTGGTGACCCTGAG	
**FW_ADAM17**	CTGGACACGTGGTTGGTGAG	NM_003183.6
**RV_ADAM17**	ATGAACAAGCTCTTCAGGTGGT	
**FW_CXCL10**	CCACGTGTTGAGATCATTGCT	NM_001565.4
**RV_CXCL10**	TGCATCGATTTTGCTCCCCT	
**FW_IL-6**	CCCACCGGGAACGAAAGA	NM_000600.5
**RV_IL-6**	TGGACCGAAGGCGCTTGT	
**FW_DYRK1A**	AAAAATCAGCGAAAGCCAGGAT	NM_001396.5
**RV_DYRK1A**	TGCTGAAGTCTCTCCTCCTGTA	
**FW_AKT**	ATGGACAGGGAGAGCAAACG	NM_005163.2
**RV_AKT**	CTGGCCACAGCCTCTGATG	
**FW_IFNB1**	GCGACACTGTTCGTGTTGTC	NM_002176.4
**RV_IFNB1**	GCCTCCCATTCAATTGCCAC	
**FW_WNT**	AAAATCCGGGGATCCTGCAC	NM_005430.4
**RV_WNT**	GTTTCTCGACAGCCTCGGTT	
**FW_NOTCH**	GACATGCCACGTGGTGGA	NM_017617.5
**RV_NOTCH**	GGCACGATTTCCCTGACCA	
**FW_AngioT**	GGGTACTACAGCAGAAGGGTATG	NM_001384479.1
**RV_AngioT**	GGGGATGTCTTGGCCTGAAT	
**FW_Renin**	GGAACAGAACTCACCCTCCG	NM_000537.4
**RV_Renin**	GTGATTCCACCCACGGTGAT	
**FW_ACE**	TCTGGCAGAACTTCACGGAC	NM_000789.4
**RV_ACE**	TTAGCAGGGCGTTGTACTGC	
**FW_SOCS4**	TGGGCACATGATGGCAGATAC	NM_199421.2
**RV_SOCS4**	CCGTCTTTTCTGTCGGCACT	
**FW_Furin**	TGGACCCCAAAATCAGCGAA	NM_002569.4
**RV_Furin**	GTGAGAGCGGTGAACCAAGA	
**FW_N-SARS**	TGGACCCCAAAATCAGCGAA	NC_045512.2
**RV_N-SARS**	GTGAGAGCGAACCAAGA	

FW, forward; RV, reverse.

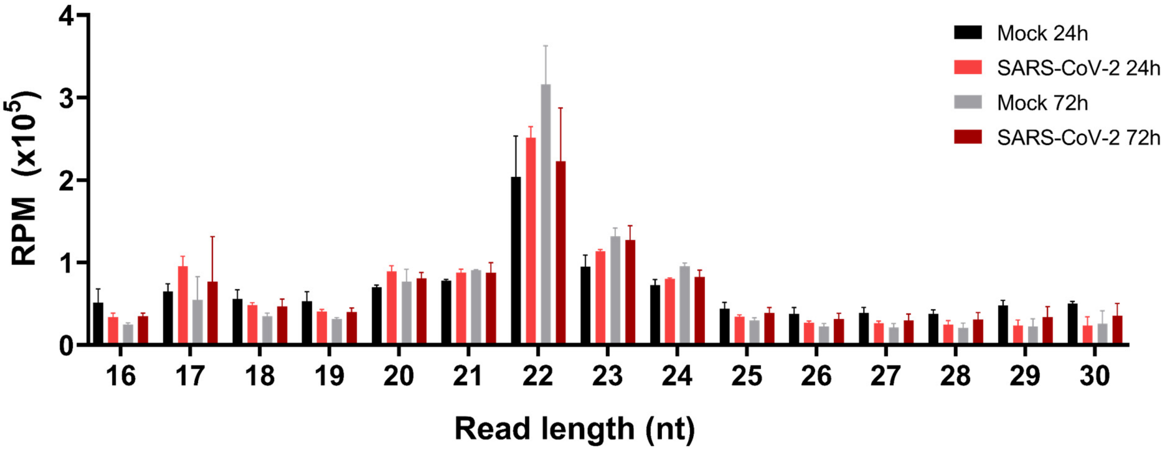

Figure S1. Adapter-trimmed reads length distribution in Calu-3 cells infected or not with SARS-CoV-2 virus. The total adaptor-trimmed read count for each length (16-30 nt) was measured for Mock and SARS-CoV-2 infected cells at 24 h and 72 h post-infection. n=3 biological replicates for each condition.

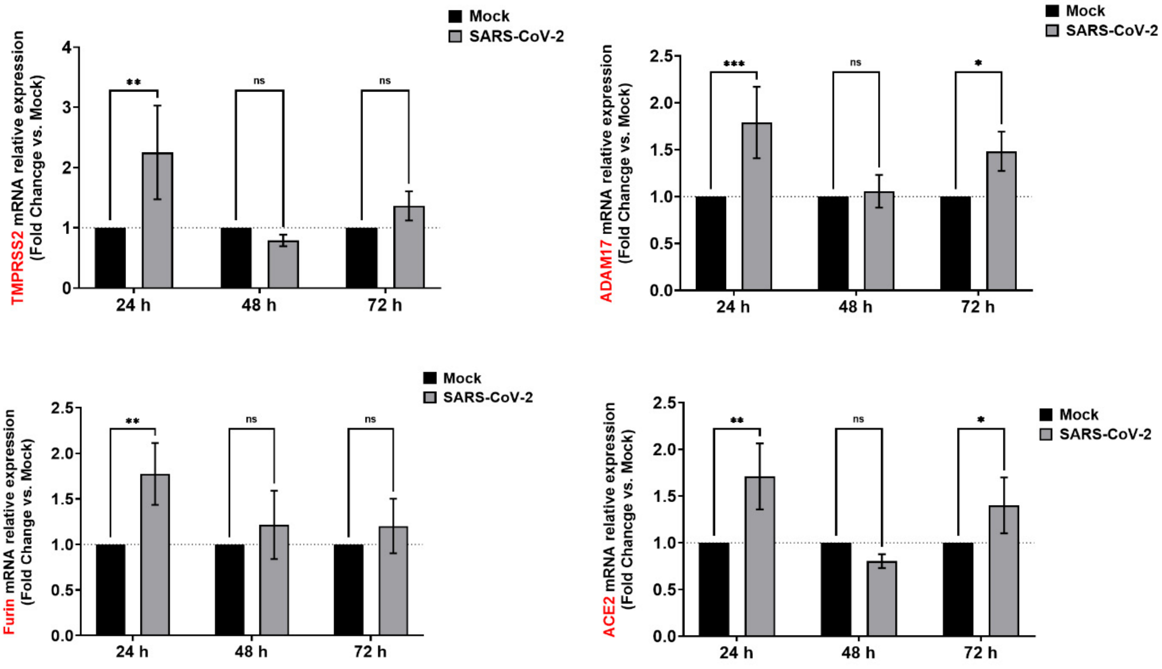

Figure S2. SARS-CoV-2 regulates host carboxypeptidases involved in the release of ACE2 ectodomain from the cell surface and in the entry of the virus into the cells. mRNA levels of *ACE2, TMPRSS2, ADAM17 and Furin* genes were monitored by RT-qPCR (relative expression) upon SARS-CoV-2 infection in Calu-3 cells. qPCR data were normalized with a reference gene (Actin beta, *ACTB*), reported to mock and expressed with a relative quantitation method (ddCT). Statistical analysis. All data presented were calculated from three biological replicates (n = 3) measurements ± SD. The ordinary two-way analysis of variance (ANOVA) and Šídák's multiple comparisons test were used for statistical analysis. Statistically significant differences (fold change vs mock) are indicated by stars (*): * $p < 0.05$; ** $p < 0.01$; *** $p < 0.001$; **** $p < 0.0001$.

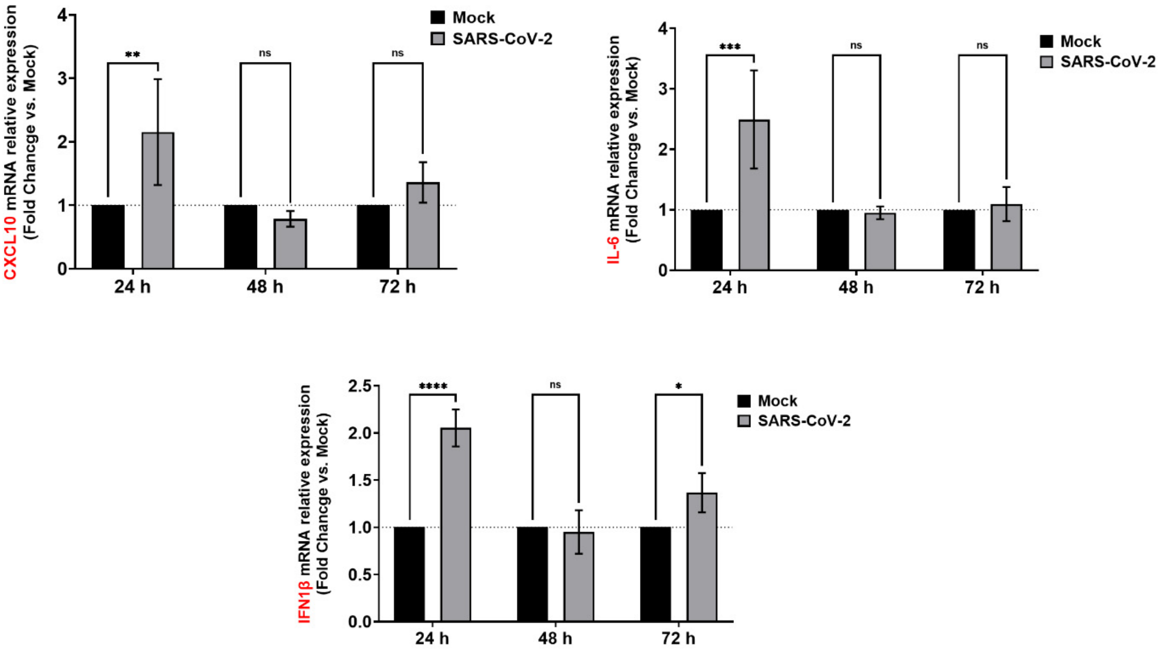

Figure S3. SARS-CoV-2 may regulate several predicted host mRNA targets involved in innate immune response. mRNA levels of *Il-6, IFN1β, CXCL10* genes were monitored by RT-qPCR (relative expression) upon SARS-CoV-2 infection in Calu-3 cells. qPCR data were normalized with a reference gene (Actin beta, *ACTB*), reported to mock, and expressed with a relative quantitation method (ddCT). **Statistical analysis.** All data presented were calculated from three biological replicates (n = 3) measurements ± SD. The ordinary two-way analysis of variance (ANOVA) and Šídák's multiple comparisons test were used for statistical analysis. Statistically significant differences (fold change vs control) are indicated by stars (*): * $p < 0.05$; ** $p < 0.01$; *** $p < 0.001$; **** $p < 0.0001$.

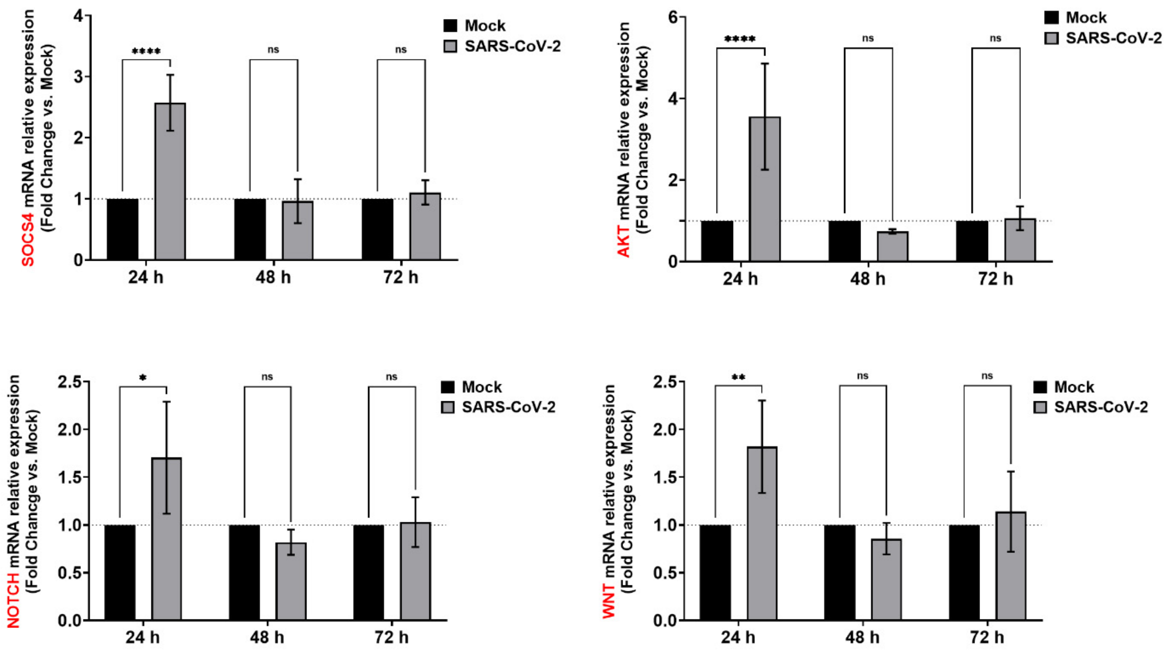

Figure S4. SARS-CoV-2 potentially regulates several predicted host mRNA targets involved in fundamental cellular processes. mRNA levels of *SOCS4*, *AKT*, *NOTCH*, *WNT* genes were monitored by RT-qPCR (relative expression) following SARS-CoV-2 infection in Calu-3 cells. qPCR data were normalized with a reference gene (Actin beta, *ACTB*), reported to control (uninfected=mock), and expressed with a relative quantitation method (ddCT). **Statistical analysis.** All data presented were calculated from three biological replicates (n = 3) measurements ± SD. The ordinary one-way analysis of variance (ANOVA) and Šídák's multiple comparisons test were used for statistical analysis. Statistically significant differences (fold change vs. control) are indicated by stars (*), * $p < 0.05$; ** $p < 0.01$; *** $p < 0.001$, **** $p < 0.0001$.

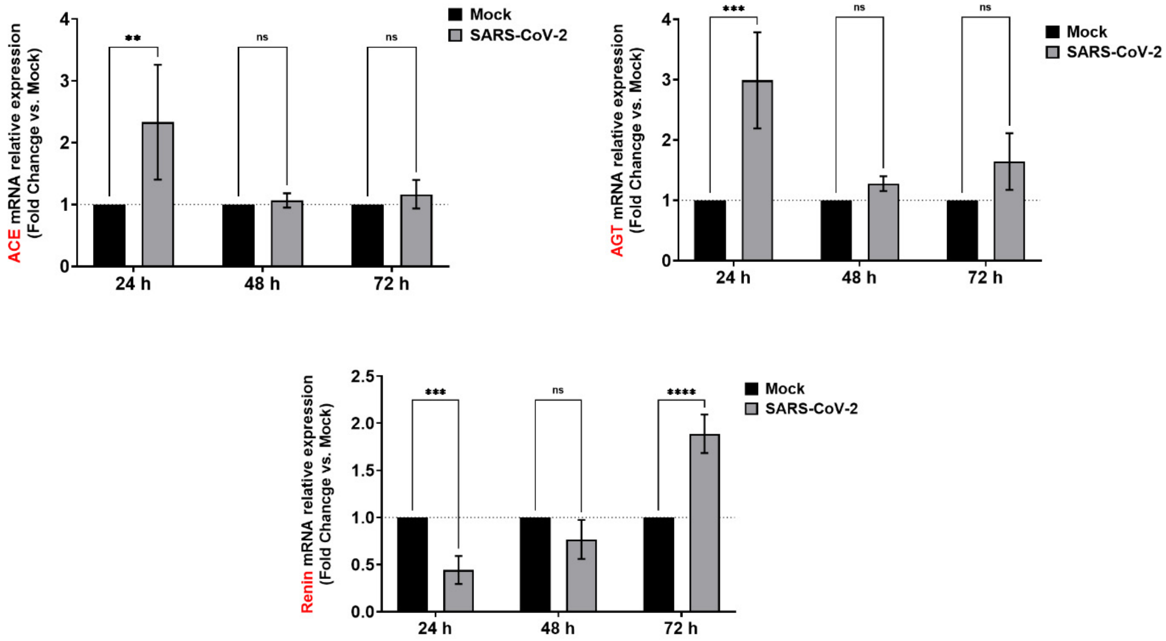

Figure S5. SARS-CoV-2 may regulate several predicted host targets involved in *Renin*-Angiotensin System (RAS). mRNA levels of *ACE, AGT* and *Renin* genes were monitored by RT-qPCR (relative expression) upon SARS-CoV-2 infection in Calu-3 cells. qPCR data were normalized with a reference gene (Actin beta, *ACTB*), reported to mock, and expressed with a relative quantitation method (ddCT). **Statistical analysis.** All data presented were calculated from three biological replicates (n = 3) measurements ± SD. The ordinary two-way analysis of variance (ANOVA) and Šídák's multiple comparisons test were used for statistical analysis. Statistically significant differences (fold change vs control) are indicated by stars (*): * $p < 0.05$; ** $p < 0.01$; *** $p < 0.001$; **** $p < 0.0001$.

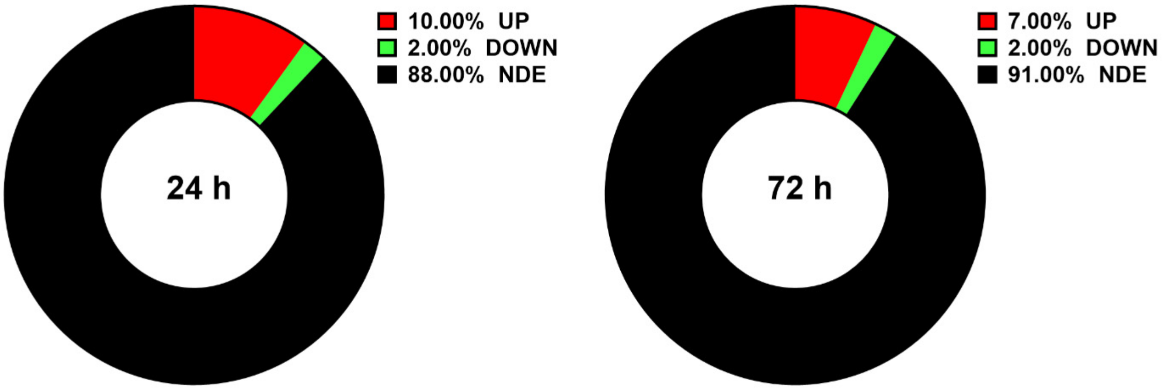

Figure S6. Proportion of differentially expressed miRNAs in Calu-3 cells infected or not with SARS-CoV-2 virus. Differentially expressed miRNAs (upregulated, downregulated, or unchanged) are extracted from the scatter plot of **Figure 6** and in reference to total number of miRNAs at 24h and 72h.

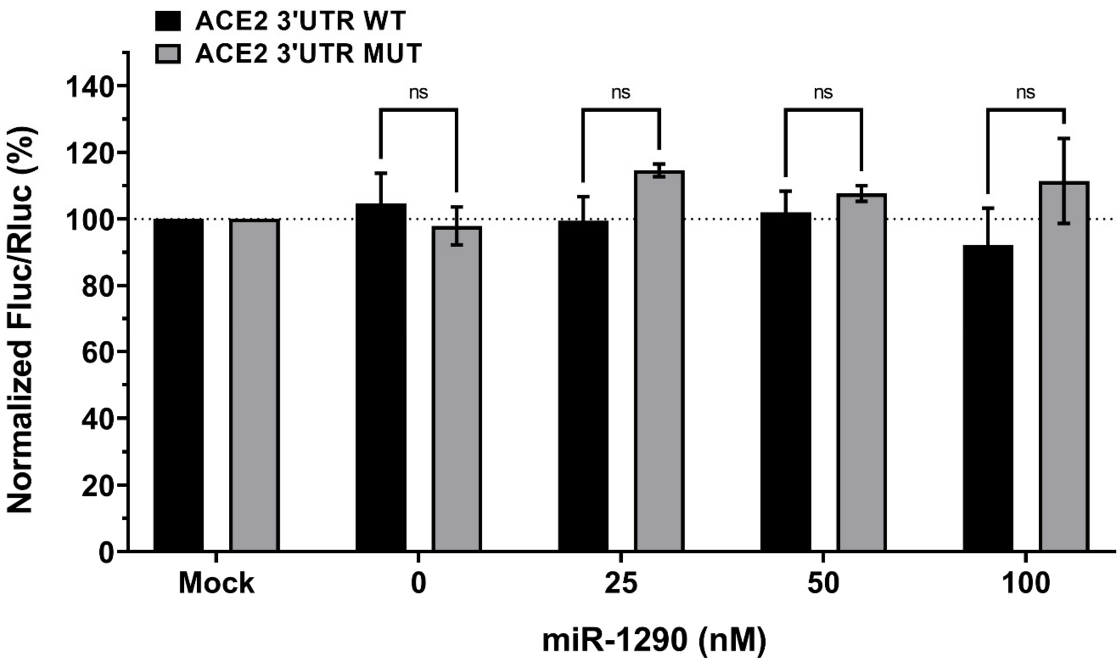

Figure S7. miR-1290 does not seem to regulate ACE2. Calu3 cells were co-transfected with homo sapiens (hsa) human miR-1290 mimic (0, 25, 50, and 100 nM) and a psiCHECK2 reporter construct (50 ng; see Supplementary File SX), in which the Rluc reporter gene was coupled with wild-type (WT) or mutated (MUT) human ACE2 3′ Untranslated Region (UTR). An unrelated, negative miRNA control (Mock) was used for normalization, in addition to the internal normalizer Fluc. "0 nM" corresponds to the transfection reagent-only control. Statistical analysis: Data were calculated from three biological replicates and expressed as means ± SD. The two-way analysis of variance (ANOVA) and Šídák's multiple comparisons test were used, and statistically significant differences (fold change WT vs. MUT) are indicated as follows: * $p < 0.05$; ** $p < 0.01$; *** $p < 0.001$; **** $p < 0.0001$; ns, nonsignificant.

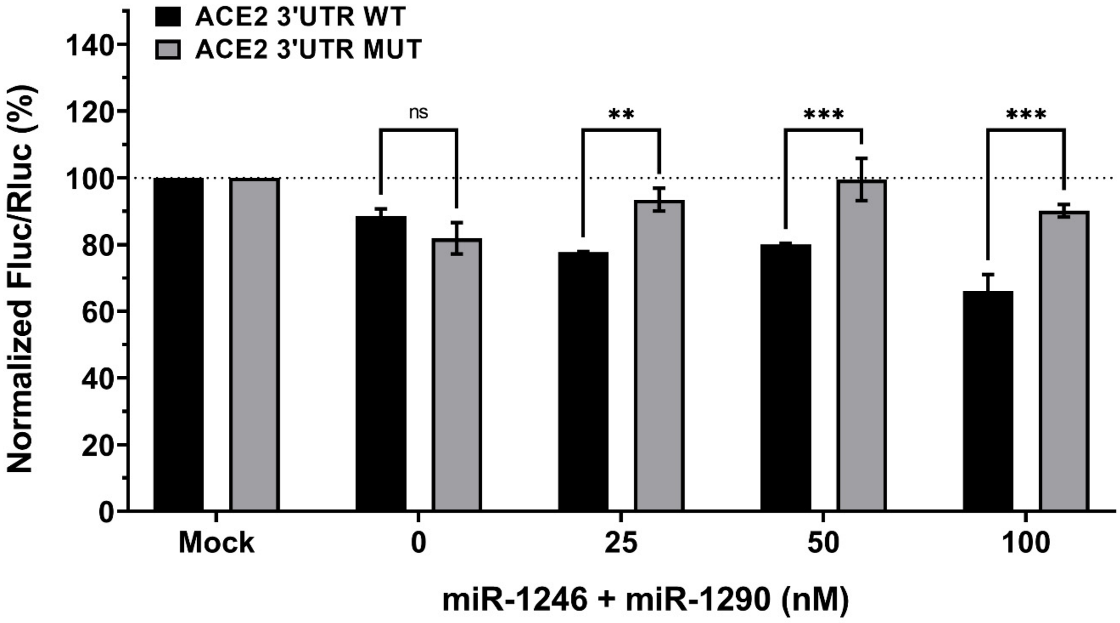

Figure S8. Combination of miR-1246 and miR-1290 did not result in a additive repressive effect on ACE2 levels. Calu3 cells were co-transfected with a mix of two homo sapiens (hsa) human miR-1246 and miR-1290 mimic (0, 25, 50, and 100 nM) and a psiCHECK2 reporter construct (50 ng; see Supplementary File SX), in which the Rluc reporter gene was coupled with wild-type (WT) or mutated (MUT) human ACE2 3′ Untranslated Region (UTR). An unrelated, negative miRNA control (Mock) was used for normalization, in addition to the internal normalizer Fluc. "0 nM" corresponds to the transfection reagent-only control. **Statistical analysis**: Data were calculated from three biological replicates and expressed as means ± SD. The two-way analysis of variance (ANOVA) and Šídák's multiple comparisons test were used, and statistically significant differences (fold change WT vs. MUT) are indicated as follows: * $p < 0.05$; ** $p < 0.01$; *** $p < 0.001$; **** $p < 0.0001$; ns, nonsignificant.

A) Upregulated miRNA targets _ SARS-COV-2 24h vs. Mock 24h

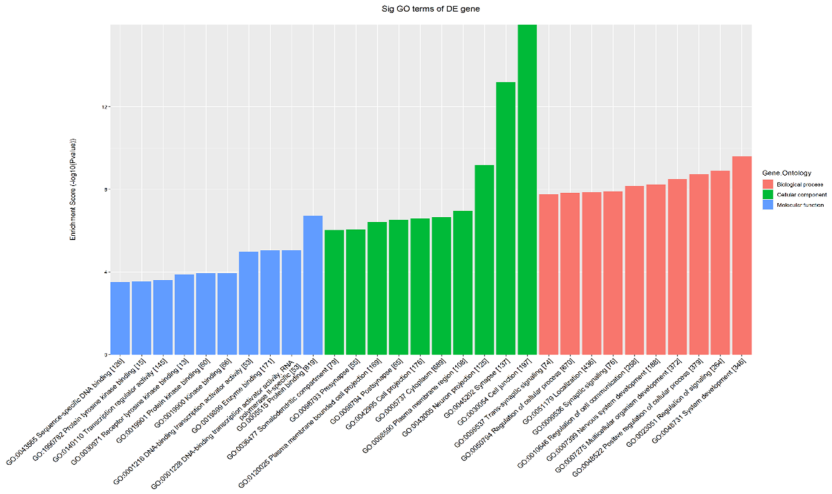

B) Upregulated miRNA targets _ SARS-COV-2 72h vs. Mock 72h

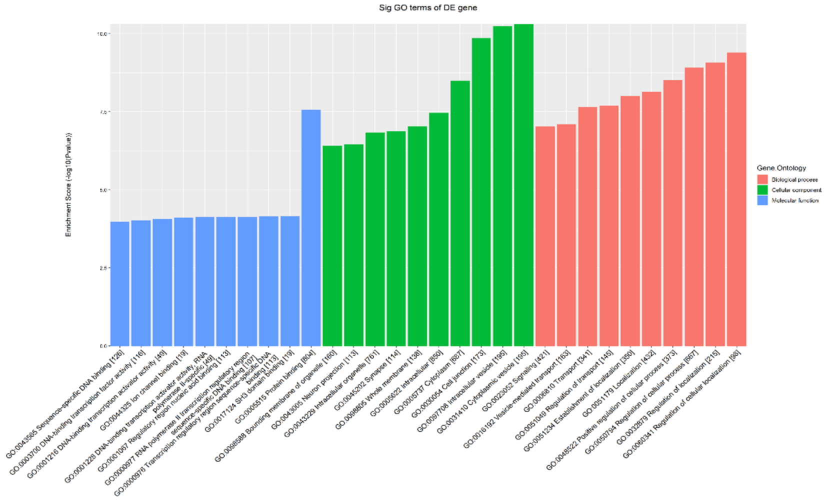

Figure S9. Enrichment score values of the top ten enrichment terms following SARS-CoV-2 infection vs. Mock. Filtered targets of upregulated miRNAs at A) 24 h and B) 72h post-infection were subjected to GO functional analysis (Biological Process, Molecular Function, Cellular Component).

A) Downregulated miRNA targets _ SARS-COV-2 24h vs. Mock 24h

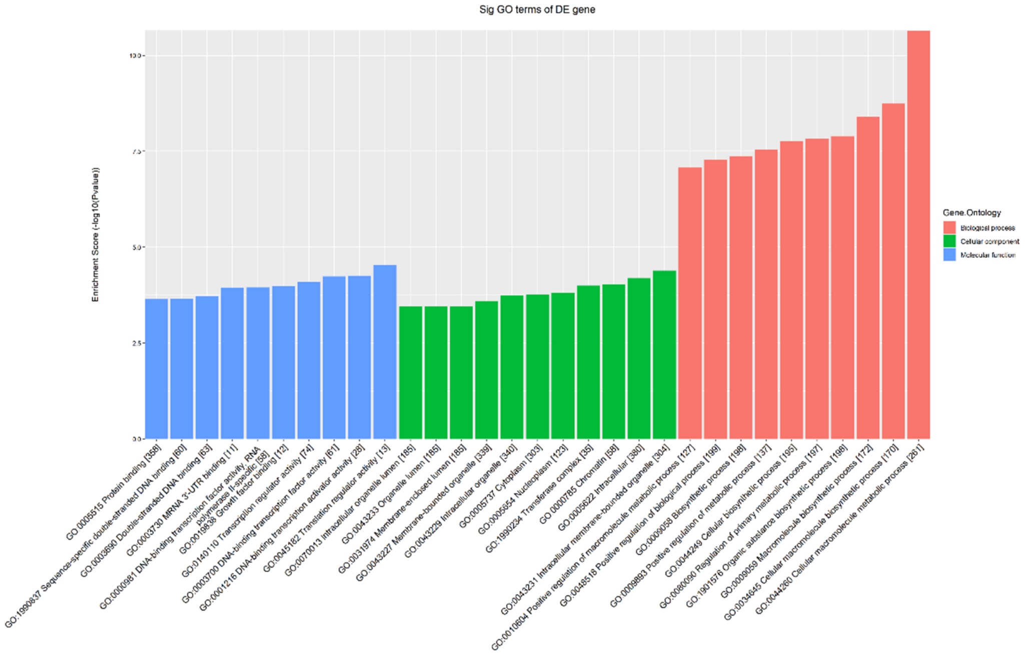

B) Downregulated miRNA targets _ SARS-COV-2 72h vs. Mock 72h

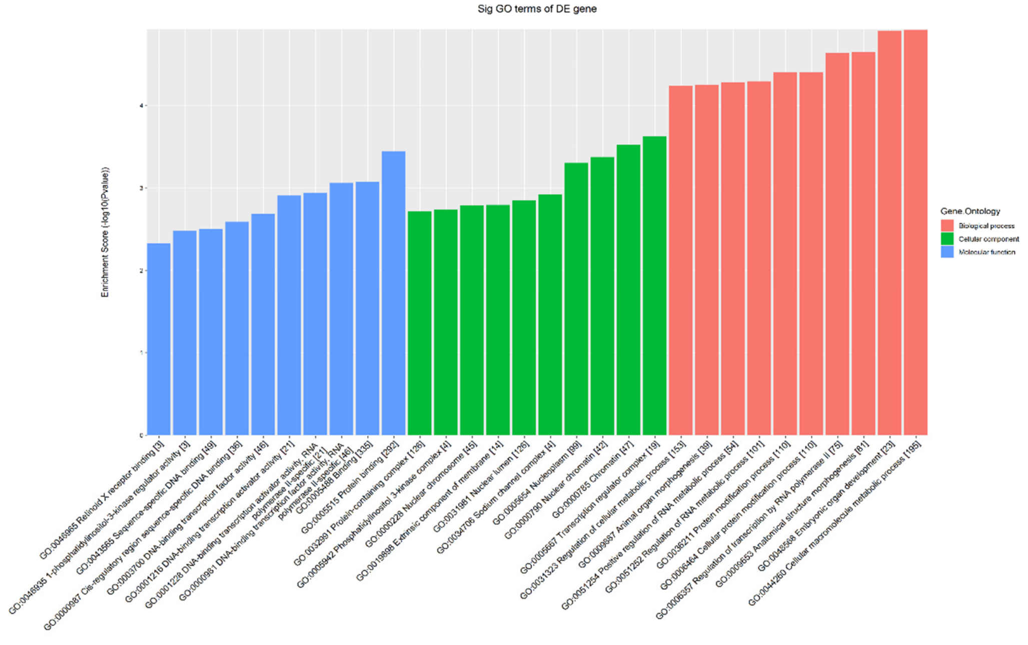

Figure S10. Enrichment score values of the top ten enrichment terms following SARS-CoV-2 infection vs. Mock. Filtered targets of downregulated miRNAs at A) 24 h and B) 72h post-infection were subjected to GO functional analysis (Biological Process, Molecular Function, Cellular Component).
